# *Gv1*, a Zinc Finger Gene Controlling Endogenous MLV Expression

**DOI:** 10.1093/molbev/msab039

**Published:** 2021-02-09

**Authors:** George R Young, Aaron K W Ferron, Veera Panova, Urszula Eksmond, Peter L Oliver, George Kassiotis, Jonathan P Stoye

**Affiliations:** 1 Retrovirus-host Interactions Laboratory, The Francis Crick Institute, London, UK; 2 Retroviral Immunology, The Francis Crick Institute, London, UK; 3 MRC Harwell Institute, Harwell Campus, Oxfordshire, UK; 4 Department of Infectious Disease, Imperial College London, London, UK

**Keywords:** *Gv1*, *Sgp3*, KRAB ZFP, TRIM28, murine leukemia virus, endogenous retrovirus

## Abstract

The genomes of inbred mice harbor around 50 endogenous murine leukemia virus (MLV) loci, although the specific complement varies greatly between strains. The *Gv1* locus is known to control the transcription of endogenous MLVs and to be the dominant determinant of cell-surface presentation of MLV envelope, the G_IX_ antigen. Here, we identify a single Krüppel-associated box zinc finger protein (ZFP) gene, *Zfp998*, as *Gv1* and show it to be necessary and sufficient to determine the $G_{\text{IX}}^{+}$ phenotype. By long-read sequencing of bacterial artificial chromosome clones from 129 mice, the prototypic $G_{\text{IX}}^{+}$ strain, we reveal the source of sufficiency and deficiency as splice-acceptor variations and highlight the varying origins of the chromosomal region encompassing *Gv1*. *Zfp998* becomes the second identified *ZFP* gene responsible for epigenetic suppression of endogenous MLVs in mice and further highlights the prominent role of this gene family in control of endogenous retroviruses.

## Introduction

Exogenous murine leukemia viruses (MLVs) and endogenous MLV loci have long been studied in mice (Stoye2012). Although few laboratory mouse strains harbor MLVs capable of replication within young, immunocompetent mice, defective proviruses can still influence immune function (Young, Ploquin, et al. 2012) and cause disease (Paprotka et al. 2011; Young, Eksmond, et al. 2012). A variety of epigenetic mechanisms thus control the expression of endogenous retroviruses (ERVs) (Yang et al. 2015), including Krüppel-associated box zinc finger proteins (ZFPs), such as Zfp809 and Zfp708 (Wolf and Goff 2009; Seah et al. 2019), which recruit Trim28 and Setdb1 to ERVs to mediate their silencing (Jacobs et al. 2014; Ecco et al. 2017; Wolf et al. 2020).

Presentation of MLV surface (SU) glycoprotein, historically the “G_IX_ antigen”, varies between strains of laboratory mice (Geering et al. 1966; Stockert et al. 1971; Obata et al. 1975; Tung, Fleissner, et al. 1975; Tung, Vitetta, et al. 1975). Crosses of $G_{\text{IX}}^{+}$ (129) and $G_{\text{IX}}^{–}$ (C57BL/6J, B6/J) strains revealed two unlinked loci controlling the phenotype, including *Gv1*, which influences tissue-specific (Levy et al. 1982) steady-state expression levels of multiple proviruses (Elder et al. 1977) and was proposed to encode a trans-acting regulatory factor with no linkage to known proviruses (Levy et al. 1985; Oliver1999). Backcrossing placed *Gv1* alongside *Sgp3* (otherwise known as *Bxs6*/*Elspg2*)(Oliver and Stoye 1999; Tucker et al. 2000; Haywood et al. 2001; Rankin et al. 2007) (fig. S1), which is analogously linked to serum gp70 levels and anti-gp70 immune responses, yet their relationship has not been conclusively addressed and the loci reportedly control different subsets of the ecotropic, modified-/polytropic, and xenotropic MLV subclasses (Oliver and Stoye 1999; Baudino et al. 2008; Yoshinobu et al. 2009; Treger et al. 2019).

Both 129-$G_{\text{IX}}^{–}$ and B6-$G_{\text{IX}}^{+}$ strains have previously been generated through serial backcrossing but have unfortunately been lost (Stockert et al. 1975). Extending recent work on *Sgp3* (Treger et al. 2019), we have now sought to formally identify the gene corresponding to *Gv1*. We determine *Zfp998* (*2410141K09Rik*) as necessary and sufficient to control the G_IX_ phenotype and identify the source of deficiency at the locus in the prototypic $G_{\text{IX}}^{+}$ strain, 129.

## Results

### Structural Variation around *Gv1*

**Fig. 1:**
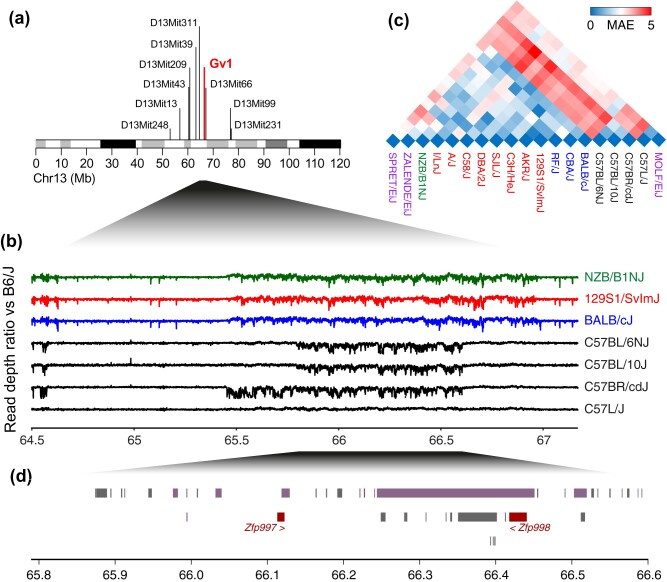
The chromosomal context of *Gv1*.(*a*) Reanalysis of a large-scale backcross (fig. S2) highlighting the location of *Gv1* on Chr 13 (GRCm38). The linkage peak was predicted by scaling predicted genetic distances to the flanking markers *D13Mit311* and *D13Mit66* to their intervening chromosomal distance. (*b*) Read depth ratios vs B6/J for exemplar strains (full data in fig. S4) showing CNVs within the area encompassing *Gv1*. C57-lineage strains are colored black and non-C57-lineage strains by phenotype: $G_{\text{IX}}^{–}$—blue, $G_{\text{IX}}^{+}$—red, and *Sgp3*^+^—green. (*c*)Heatmap of the median absolute error (MAE) between read depth ratio profiles for the strains in fig. S4. All non-C57-lineage strains group with ZALENDE/EiJ, whereas the C57-lineage group separately, and C57L/J with MOLF/EiJ. Coloring is according to (*c*), with wild-derived inbred strains in purple. (*d*) View of the genes within the area deleted within B6/N mice. Pseudogenes are colored gray, lincRNAs in purple, and protein coding genes in maroon. The protein coding ZFPs are annotated.

Given *Gv1*’smapping preceded publication of the mouse genome, we first reanalyzed previously-acquired large-scale backcross data (Oliver1999) to confirm its location. Assessment of nine markers across a 1,108-animal backcross (fig. S2) refined its location to the region between *D13Mit311* and *D13Mit66* (13:63.64–67.17 Mb) and identified peak linkage at 13:66.58 Mb (fig. 1*a*), within a large cluster of ZFPs (Kauzlaric et al. 2017). ZFP clusters form through local gene duplication events (Huntley et al. 2006) and extensive self-homology complicates their analysis (fig. S3); indeed, although many variations have been reported within this area (Henrichsen et al. 2009; Quinlan et al. 2010; Keane et al. 2011; Pezer et al. 2015), no two studies identified shared differences or grouped inbred strains consistently. Similarly, Mouse Genomes Project variant calls (Keane et al. 2011; Yalcin et al. 2011) revealed no consistent differences between $G_{\text{IX}}^{+}$ and $G_{\text{IX}}^{–}$mouse strains or between *Sgp3*^+^ and *Sgp3*^–^strains within the candidate region.

However, read depth analysis revealed surprising variation in comparison to B6/J. Most strains shared a ∼1.49 Mb region (13:65.47–66.96 Mb) of apparent copy-number variation (CNV) irrespective of their G_IX_ or *Sgp3* status (fig. 1*b*, fig. S4). Notably, C57-lineage strains (littermates of Lathrop’s F57 × M52 cross [Beck et al. 2000]) were distinct, although only C57L/J mice were equivalent to B6/J, as an ∼810 kb deletion was evident in C57BL/10J and C57BL/6N (B6/N) (13:65.79–66.60 Mb), which extended to ∼1.15 Mb in C57BR/cdJ (13:65.45–66.60 Mb). In comparison, areas of both increased and reduced depth within the non-C57-lineage strains was indicative of an alternate chromosomal conformation, potentially reflecting known differences in the contributions of different subspecies to the genomes of laboratory mice (Yang et al. 2007, 2011). Read depth profile clustering supported this theory, grouping C57-lineage mice with *Mus musculus molossinus* (MOLF/EiJ) and non-C57-lineage strains with *M. m. domesticus* (ZALENDE/EiJ) (fig. 1*c*, fig. S4). This was separately confirmed using the Mouse Phylogeny Viewer (Yang et al. 2011) (fig. S5). Importantly, non-C57-lineage strains also grouped with *Mus spretus* (SPRET/EiJ), which shares a common ancestor with the subspecies of *M. musculus* ∼2.3 million years ago and represents the ancestral state of the chromosome (Kumar et al. 2017).

Thus, the origin of the chromosomal region encompassing *Gv1* differs between the prototypic $G_{\text{IX}}^{–}$ strain, B6/J, and the majority of other laboratory strains. Additionally, several C57-lineage strains displayed large, unreported, deletions within the candidate region.

### *Zfp998* is Necessary and Sufficient to Determine G_IX_ Status

**Fig. 2:**
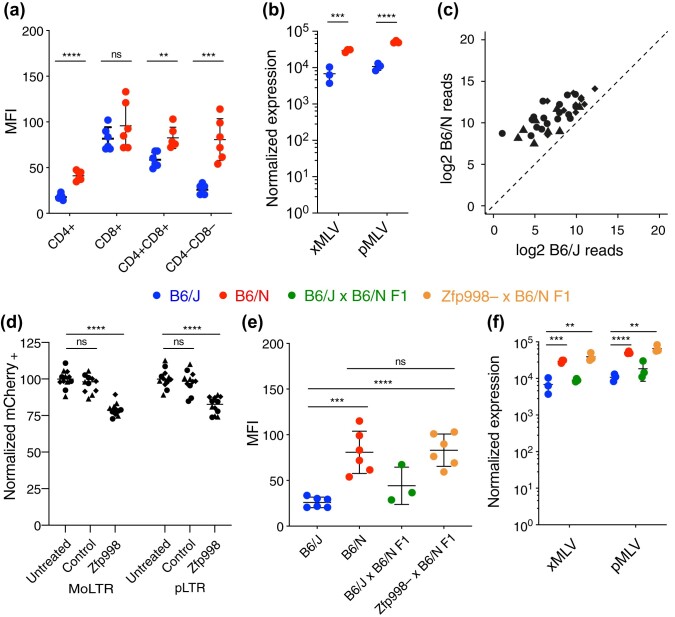
Zfp998 controls endogenous MLV expression *in vitro* and *in vivo*.(*a*) Median fluorescence intensity of 83A25 staining for MLV SU within the indicated subsets of CD19^–^ thymocytes (six mice per group). (*b*) expression of x- and pMLV *envelope* measured by qRT-PCR and normalized relative to *Hprt* (three mice per group). Amplification of products for e- and mpMLV was negligible or absent after 40 cycles. (*c*)RNAseq expression analysis of endogenous MLVs in B6/N and B6/J (three mice per group). Significantly (*q* < 0.01, log2FC > 1) regulated MLV loci are indicated, with symbol shape denoting MLV subclass: triangle—mpMLV, circle—pMLV, and diamond—xMLV. (*d*) Numbers mCherry^+^ cells in otherwise untreated 293 T cells, the same cells transfected with a control plasmid, or with a *Zfp998*-containing expression plasmid. Symbol shape denotes the results of three independent experiments with 3/4 technical replicates. Significance values are from two-way ANOVA comparisons with no significant differences being determined between experiments or in experiment: treatment interactions. (*e*) Median fluorescence intensity of 83A25 staining for MLV SU within the indicated mouse genotypes (six, six, three, and six mice per group). (*f*)Expression of x- and pMLV *envelope* measured by qRT-PCR and normalized relative to *Hprt* (three mice per group). Amplification of products for e- and mpMLV was negligible or absent after 40 cycles. In (*a*), (*b*), (*e*), and (*f*), significance values are from Student’s t-tests with the indicated group sizes. ** = *p* < 0.01, *** = *p* < 0.001, **** = *p* < 0.0001.

As the deletion within B6/N (fig 1*b*) fell within the area defined by the fine mapping of *Gv1* (fig. 1*a*), we sought to determine the G_IX_ status of this strain. Reflecting G_IX_’s historical usage as a T-cell marker (Obata et al. 1975), we stained for MLV SU across different populations of CD19^–^thymocytes (fig. S6). Significant differences between B6/N and B6/J were observed for three populations (fig. 2*a*) and most substantially for CD4^–^CD8^–^ thymocytes. For a more holistic view, we confirmed this for whole-spleen samples by qRT-PCR (fig. 2*b*), where both pMLV and xMLV loci displayed significant upregulation, and by RNAseq, which revealed 14.5- and 5.3-fold average upregulation of the same groups, respectively (fig. 2*c*). No further significant stratifications, including on tRNA primer binding site (PBS) usage, were visible.

This placed *Gv1* within the ∼810 kb deletion in B6/N, within which only two genes encoding KRAB-containing ZFPs are annotated: *Zfp997* and *Zfp998*, with 19 and 14 zinc fingers, respectively (fig. 1*d*). These genes also formed the candidates for *Sgp3* (Treger et al. 2019) and, although explicit identification was precluded by lack of separation in knockout animals, *Zfp998* alone displayed specificity for the MLV LTR in electrophoretic mobility shift assays and was thus suggested as the most likely candidate. As *Zfp998* was also the closest annotated protein-coding *ZFP* gene to the predicted point of peak linkage for *Gv1* (fig. 1*a*), falling only 142 kb proximally, we chose to confirm its ability to control the expression of reporter-bearing MLV proviruses in a cell-based system, which was not previously assessed. Transient expression of *Zfp998* significantly reduced numbers of mCherry^+^ cells in cultures bearing integrated viral genomes driving the reporter from either a Moloney-MLV or a consensus pMLV LTR (20.5% and 18.3% versus untreated controls, respectively) (fig 2*d*). This was equivalent to the difference in MLV SU staining observed for the CD8^+^ and CD4^+^CD8^+^ thymocyte populations (15.3 and 29.0%, respectively) (fig. 2*a*) and similarly representative of the 26–59% reductions seen upon *Zfp708* treatment of RMER19B LTRs (Seah et al. 2019).

To next determine if individual disruption of *Zfp998* was sufficient to recreate the $G_{\text{IX}}^{+}$ phenotype in B6/J, we created knockout mice using CRISPR/Cas9 targeting. Two independent disruptions of the gene were confirmed by copy-number, split-read, and split-mate analyses of 10× PE150 whole genome sequencing (WGS) data, one truncating exon 4 (containing the zinc fingers) and one removing exons 1–3 (fig. S7). Importantly, no disruption of *Zfp997* was noted in either case. Subsequent investigations of G_IX_ phenotype were conducted using levels of MLV SU cell-surface staining and qRT-PCR. Within the control groups, as before, significantly higher levels of MLV SU were observed for B6/N than for B6/J mice (fig. 2*e*), which was reflected in elevated xMLV and pMLV expression (fig. 2*f*). Consistent with *Gv1*’s semi-dominant mode of action (Stockert et al. 1971), non-significant increases were also visible in B6/J × B6/N F1s. In comparison to this latter group, however, B6/J-*Zfp998*^–^× B6/N F1s fully recapitulated the $G_{\text{IX}}^{+}$ phenotype of B6/N, both by MLV SU staining and qRT-PCR. Identical results were observed for both forms of the knockout, which are presented together.

### Resolving the Source of *Zfp998* Insufficiency

Together, these data confirmed that loss of Z*fp998* was singularly sufficient to confer a $G_{\text{IX}}^{+}$phenotype within the B6 background and that its expression reduced expression of MLV proviruses in a biological system. Nevertheless, its chromosomal context differs substantially between C57- and non-C57-lineage laboratory mice and, amongst these, no groupings were visible that could define G_IX_ status (fig 1*c*, fig. S4). Similarly, manual assessments of mouse genealogies (Beck et al. 2000) revealed no links in the origins of $G_{\text{IX}}^{+}$ or $G_{\text{IX}}^{–}$ strains.

**Fig. 3:**
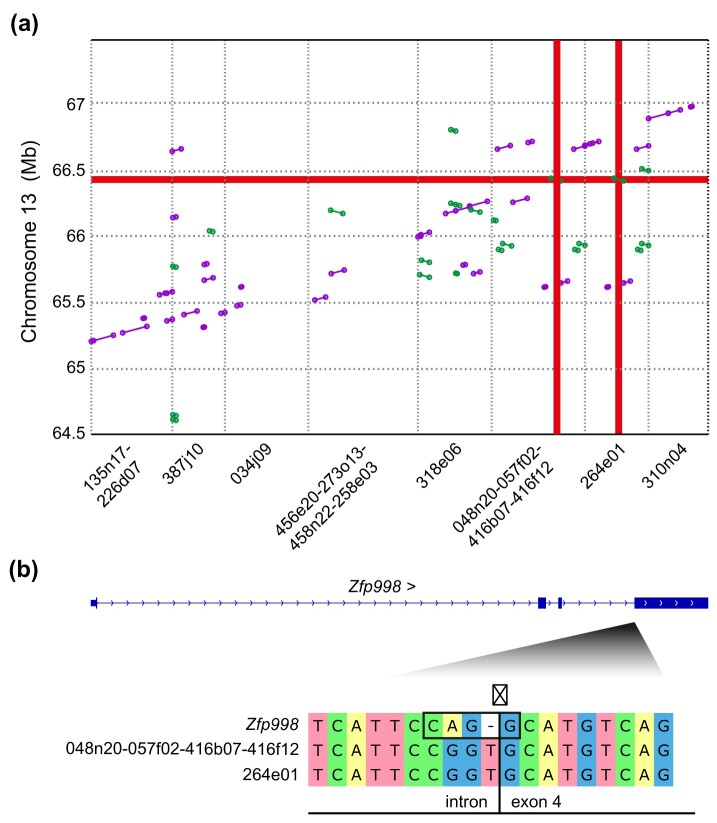
Nanopore sequencing allows assembly of the *Gv1* gene region from 129 mice.(*a*) Alignment of the eight contigs resulting from assembly of the BACs against Chr 13. Diagonal lines represent only those areas ≥1 kb with ≥98% identity to the GRCm38 reference and are colored according to orientation (purple—forwards, green—reverse). Sequence names detail the scaffolding order and final sizes are shown above the alignment sections. Red highlighting shows the single position of *Zfp998* in C57BL/6J (horizontal) intersecting with two independent copies within the BAC assemblies (vertical). (*b*) Comparison of the splice acceptor sequence (black box, with arrow marking point of splicing) for exon 4 of *Zfp998* in comparison to the equivalent gene regions from the two assembled scaffolds. Area shown is the reverse complement of GRCm38 13:66432223-66432240.

We therefore determined to clarify the status of the *Gv1* locus within the prototypic $G_{\text{IX}}^{+}$ strain, 129, which displays amongst the highest levels of cell-surface MLV SU (Stockert et al. 1971) and for which the AB2.2 ES cell line-derived bMQ bacterial artificial chromosome (BAC) library provides an excellent resource for comparative genetics (Adams et al. 2005). Sixteen BAC clones predicted to span the region were purchased, amplified in culture, and isolated using techniques to maintain their full lengths for subsequent Oxford Nanopore MinION long-read sequencing. This allowed for high coverages (median 2494×) to be achieved using only ≥1 kb reads and the full and contiguous assembly of all 16 BACs (fig. S8). Upon removal of pBACe3.6 vector sequences, 15 BAC sequences could be joined into eight scaffolds that aligned to the region (fig. 3*a*). Although a single scaffold could not be resolved, precluding orientation in relation to the B6/J-based GRCm38 reference and determination of the quantity of missing sequence between the areas assembled, large areas of sequence duplication (multiple *X*-*Y* paths occurring over the same *Y* axis ranges) were visible, as well as areas of unique sequence (*X* axis ranges with no corresponding *X*-*Y* path).

Surprisingly, *Zfp998* appeared not only present but apparently existed as two independent copies within the scaffolds (fig. 3*a*), both displaying 100% identity to the coding region of the *Zfp998* reference. However, both exhibited defects at the splice acceptor for the terminal, zinc-finger-containing, exon, lacking the consensus mammalian splice acceptor sequence (N[C/T]AG↓G) (fig. 3*b*). Both variations in comparison to the *Zfp998* reference sequence are classified as “high” impact splice acceptor variants by the Ensembl Variant Effect Predictor (McLaren et al. 2016) and would be predicted to occlude canonical splicing and production of functional protein. Full characterization of expression and splicing patterns for the identified loci would require complete, contiguous, assembly of the alternate chromosomal region derived from *M. m. domesticus*, as the level of variation observed (fig. 1*b*, fig. S4) otherwise precludes the non-spurious mapping of RNAseq data from non-C57-lineage strains to the B6/J-based mouse reference across this region.

Together, these data account for the prototypic G_IX_positivity and negativity of 129 and B6/J, respectively. Likely through tandem duplication, the majority of laboratory mice contain two copies of the area in which *Zfp998* exists within B6/J, although neither harbor functional copies of the gene in its reference state. Present in *M. m. domesticus* and *M. spretus*, this configuration represents the ancestral state within *Mus*. In contrast, the equivalent region from *M. m. molossinus*, present within B6/J, contains a single copy of the gene region that has been modified to allow splicing into the terminal exon, thereby creating a functional *Zfp998* gene.

## Discussion

The means by which hosts control the expression and, ultimately, the mobility and pathogenicity of endogenous repetitive elements is of great interest. Work detailing the complement and expression patterns of endogenous MLVs stems from the original creation of inbred lines by and for mouse fanciers, where the first detailed record-keeping allowed investigation of so-called “heritable cancers”(Russell1985). Following characterization of the G_IX_ phenotype in the 1960s, the *Gv1* locus was identified in 1971 (Stockert et al. 1971), although early mapping attempts were contradictory (Stockert et al. 1976) and the locus was only later localized to chromosome 13 (Oliver and Stoye 1999).

An analogous locus, *Sgp3* was recently identified as either *Zfp998* (dubbed *Snerv1*) or *Zfp997* (*Snerv2*) (Treger et al. 2019) and we now positively identify *Zfp998* as *Gv1*, making it highly probable that *Gv1* and *Sgp3* are differing phenotypic readouts of the same underlying gene, related to the specific mouse strains in which they have been studied. As such, it may be concluded that previously reported distinctions in the subclasses of MLVs regulated by the two loci likely reflect the differing complements of proviruses between strains—indeed, only ∼1/3 of MLV loci are commonly shared between any two backgrounds (Frankel et al. 1990), making this highly plausible—and we note that our data support a wider action for *Gv1* than previously reported (Oliver and Stoye 1999).

Our work predicts that *Zfp998* insufficiency is shared amongst non-C57-lineage mice and explains why the majority exhibit G_IX_positivity, albeit to varying degrees (Stockert et al. 1971). Amongst the few non-C57-lineage strains historically genotyped as $G_{\text{IX}}^{–}$, it is further possible that negativity results from a lack of expression-competent proviruses and/or suitable transcriptional milieus, rather than from *Zfp998* sufficiency. An exception must be seen in BALB/c mice; however, which also display linkage to the *Gv1* locus in crosses to 129 (Oliver and Stoye 1999), suggesting that further work with the locus may yield additional insights. Additional research may also be required to clarify the mechanism of Zfp998-based control. In contrast to the previous identification of PBS^Gln1^ as the binding site for Zfp998(Treger et al. 2019), our data suggest that the promoter activities of LTRs bearing either PBS^Gln1^ or PBS^Pro^ are repressed to equivalent, physiological, extents.

This research highlights the difficulty of studying genes influencing multiple, insertionally-polymorphic, loci and underscores the necessity of working on defined backgrounds. High levels of homology within ZFP clusters hinder application of established wet- and dry-lab techniques and there is strong potential that further uncharacterized differences have significant bearings on inter-strain diversity. We note that even within the exceptionally well-characterized B6 substrains, we have revealed variations completely unexpected in magnitude that have eluded previous bioinformatic studies of the same WGS datasets (Simon et al. 2013). Overcoming these problems, we confirm another ZFP-based control of MLV expression alongside *Zfp809*(Wolf and Goff 2009), further highlighting the importance of epigenetic controls in the establishment of ERV repression.

## Materials and Methods

Full methods are available as Supplementary Information.

## Data Availability

All raw sequencing data generated in this study have been submitted to the European Nucleotide Archive under accessions PRJEB40145 and PRJEB40276.

## Supplementary Material

msab039_Supplementary_Data
