# Supplementary material for: Gv1, a Zinc Finger Gene Controlling Endogenous MLV Expression
Source: Mol Biol Evol. 2021 Feb 9;38(6):2468–74. doi: 10.1093/molbev/msab039 (PMC8136514; doi:10.1093/molbev/msab039)
Supplement: msab039_Supplementary_Data [file msab039_supplementary_data.zip › Fig S8.pdf]

123kb **226d07**

108kb **387j10**

171kb **034j09**

106kb **258e03**

90kb **416b07**

126kb **048n20**

131kb **264e01**

90kb **416f12**

90kb **057f02**

98kb **273o13**

144kb **456e20**

78kb **094c11**

122kb **310n04**

102kb **458n22**

152kb **135n17**

151kb **318e06**
