# Supplementary material for: Gv1, a Zinc Finger Gene Controlling Endogenous MLV Expression
Source: Mol Biol Evol. 2021 Feb 9;38(6):2468–74. doi: 10.1093/molbev/msab039 (PMC8136514; doi:10.1093/molbev/msab039)
Supplement: msab039_Supplementary_Data [file msab039_supplementary_data.zip › Gv1_Supplementary_Figure_Legends.docx]

**Figure S1. Linkage of *Gv1*, *Sgp3*, *Bxs6*, and *Elsgp2* to chromosome 13.** LOD scores compiled from published data (Oliver and Stoye 1999; Tucker et al. 2000; Haywood et al. 2001; Rankin et al. 2007) with locations of all markers updated to reflect their current positions within GRCm38. *Sgp3*, *Elsgp2*, and *Bxs6* are now considered the same locus but were originally mapped independently and all three mappings are shown for reference.

**Figure S2.** **Reanalysis of a large-scale backcross locating *Gv1*.** Tabulated results of selected markers from a 1,108-animal B6/J × 129 backcrossing experiment for *Gv1*. Black and dotted shading representing animals of G_IX_^-^ and G_IX_^+^ phenotypes, respectively, and numbers with the represented genotypes are shown at the base of each column. Predicted genetic distances to each marker from the genotype frequencies are shown and are highlighted in bold text if reciprocal genotypes were identified within the backcross experiment or in italics if only one of the pair was identified.

**Figure S3. Self-alignment of 13:64.5-67.2 Mb.** Self-alignment of the area encompassing *Gv1* within GRCm38, with diagonal lines showing the positions of regions ≥1 kb with ≥98% identity. Coloring indicates the orientation of the homology: purple – forwards, green – reverse.

**Figure S4.** **Copy number variation around *Gv1*.** Data from Fig 1b for the area of GRCm38 encompassing *Gv1* alongside a larger number of other strains, colored identically. Additional wild-derived inbred strains are shown in purple. Visible is the grouping of non-C57-lineage strains, regardless of phenotype, with each other and with ZALENDE/EiJ and SPRET/EiJ. C57L/J resembles MOLF/EiJ, whereas other C57-lineage strains display deletions within the region. The relative similarity of the profiles shown are displayed in Fig 1c.

**Figure S5. Origins of the *Gv1* candidate region.** Data from the Mouse Phylogeny Viewer (Yang et al. 2011), ordered and with names colored to match Fig S4. Blue regions represent regions deriving from *M. m. domesticus*, red regions haplotypes from *M. m. musculus*, and white areas of undetermined origin. Visible amongst C57-lineage strains is a ~6.24 Mb region (GRCm38 13:63.81–70.05 Mb) deriving from *M. m. musculus*.

**Figure S6.** **FACS gating strategy.** Gating methodology for FACS of CD19^–^ thymocytes.

**Figure S7. Diagram of CRISPR/Cas9 deletions.** Two CRISPR/Cas9-mediated deletions were identified that originated from the targeted site within exon 4 and spanned ~21 and ~24 kb in either direction, as indicated, although 10× PE150 WGS did not allow their base-level resolution given the complexity of mapping accurately within ZFP clusters. Positions are indicated relative to the GRCm38 reference.

**Figure S8. BAC assemblies.** Assembled sizes (nearest kb) of the 16 BACs selected for sequencing.
