# Supplementary material for: Gv1, a Zinc Finger Gene Controlling Endogenous MLV Expression
Source: Mol Biol Evol. 2021 Feb 9;38(6):2468–74. doi: 10.1093/molbev/msab039 (PMC8136514; doi:10.1093/molbev/msab039)
Supplement: msab039_Supplementary_Data [file msab039_supplementary_data.zip › Gv1_Supplementary_Information.docx]

***Gv1*, a zinc finger gene controlling endogenous MLV expression**

George R Young, Aaron K W Ferron, Veera Panova, Urszula Eksmond, Peter L Oliver, George Kassiotis, Jonathan P Stoye

**Methods**

Mice

All animal experiments were approved by the Ethical Committee of the Francis Crick Institute and conducted according to local guidelines and UK Home Office regulations under the Animals Scientific Procedures Act 1986. Inbred C57BL/6J and C57BL/6NTac mice were maintained at the Francis Crick Institute’s animal facilities under specific pathogen-free conditions and used between 8 and 12 weeks of age. *Zfp998*-deficient C57BL/6J mice were created by CRISPR/Cas9-mediated homology-directed repair (HDR). Briefly, a plasmid encoding Cas9 was electroporated together with the *Zfp998*-targeting guide RNA (sgRNA) 5’-TTTGATGTTGGAGATGAG-3’ targeting exon 4 of the protein and the repair template 5’-GGAGATGACTGAGCCTTGCAAAGGCTTTCCCACATTGATTACATTCATAAGGTTTCTCTCCAGTATGGGTTCTTTTGATGTTGGAGATGAGTGGGTCTTGCAAAGGCTTTACTACATTGATTACATTCATATGGTTTCTCTCCAGTATGGGTTCTTTTATGACATTGGAGATGACTGGGT-3’, designed to ensure frame-shift of the exon, into fertilized C57BL/6J oocytes. Pups were genotyped by Transnetyx (Memphis, USA) and genotypes verified by 10x WGS. Targeted mice were crossed to C57BL/6N mice, in which *Zfp998* is deleted through natural structural variation, to obtain F1 progeny carrying either one copy of the WT or CRISPR/Cas9-targeted allele of the C57BL/6J parent.

Backcross data re-analysis

Primer sets for the markers previously utilized (Oliver 1999) were checked for off-target binding with Primer-BLAST and accurate positions recorded for those passing this filter. Data from the 1,108-animal backcross was re-assessed for the 9 markers retained and LOD scores calculated using standard methodologies.

FACS analysis

Single-cell suspensions were prepared by mechanical disruption of tissues through 70 μm nylon filters (Thermo Fisher, Waltham, USA). ﻿MLV SU was detected using the cross-reactive biotinylated primary 83A25 rat IgG2a mAb (Evans et al. 1990), followed by APC conjugated streptavidin (Invitrogen (Thermo Fisher), Waltham, USA), as previously described (Young et al. 2012). Co-stainings were performed with anti-CD3 (FITC, clone 145-2C11), -CD4 (eFluor 450, clone RM4-5), -CD8 (PerCP Cy 5.5, clone 53-6.7), and -CD19 (PE, clone MB19-1), purchased from BioLegend (San Diego, USA). Staining was performed for 30 mins at room temperature and samples analyzed on Fortessa cell analyzers (Becton Dickinson, Franklin Lakes, USA). Data were analyzed and plotted with FlowJo 10 (TreeStar, Ashland, USA).

qRT-PCR MLV expression analysis

﻿MLV RNA expression assessment by qRT-PCR was conducted using primers and techniques previously described (Young et al. 2012). Briefly, RNA was extracted using the RNeasy Mini QIAcube Kit (Qiagen, Hilden, Germany), treated with DNaseI (Qiagen), and used to produce cDNA with the High-Capacity Reverse Transcription kit (Applied Biosystems (Thermo Fisher), Waltham, USA) with added ribonuclease inhibitor (Promega, Madison, USA). cDNA was cleaned through QIAquick PCR purification columns (Qiagen) and eluted in 200 μL nuclease-free water (Qiagen). 1 µL of sample is used in each 25 µL qRT-PCR reaction using Fast SYBR Green (Applied Biosystems) on QuantStutio 3 instruments (Applied Biosystems). Data are analyzed relative to *Hprt* using the 2^ΔΔCt^ method. Tests for normality, followed by unpaired 2-tailed Student’s t-tests were conducted within Prism 9 (GraphPad, San Diego, USA) and plots prepared within the same software.

RNAseq MLV expression analysis

DNaseI treated RNA from samples of spleen tissue (see above) were alternatively prepared and sequenced with a PE100 chemistry. Reads were clipped of sequencing adapters and quality trimmed using Trimmomatic 0.32 (Bolger et al. 2014) and aligned to the GRCm38 reference using HISAT2 2.0.1b/2.0.4 (Kim et al. 2015). Eliminating multi-mapping reads, gene expression estimates were produced with featureCounts (Subread 1.4.6-p1) (Liao et al. 2014) for Ensembl genes (GRCm38.78) and repetitive element features produced as previously described (Attig et al. 2017). Differential expression analyses were conducted with DESeq2 (Love et al. 2014) and data plotted within R.

bMQ BACs

Raw end-read data generated with T7 and SP6 primers from within the pBACe3.6 vector were downloaded from NCBI TraceDB, base-called and trimmed using phred 0.071220c (Ewing et al. 1998; Ewing and Green 1998), and aligned to GRCm38 using GMAP 2017-03-17 (Wu and Watanabe 2005). A subset of clones where both reads mapped concordantly (inwards facing reads mapping to the same chromosome and separated by ≤500,000 bp) was prepared and 16 clones ordered from Source BioScience (Nottingham, UK) that were predicted to span the *Gv1* locus. Clones were re-streaked onto LB agar supplemented with 20 μg/mL chloramphenicol and individual colonies picked for upscaling. High molecular weight, intact, DNA was prepared from cultures with the Qiagen Large-Construct Kit, which includes an exonuclease digestion step to remove contaminating genomic DNA and sheared BAC DNA.

MinION sequencing

BAC DNA was prepared for sequencing using the SQK-RBK004 Rapid Barcoding Kit (Oxford Nanopore Technologies, Oxford, UK) according to the manufacturer’s guidelines and sequenced on two FLO-MIN106 flow cells (Oxford Nanopore Technologies). Reads were base-called and demultiplexed with Albacore 2.3.1 (Oxford Nanopore Technologies), assembled with canu 1.7.1 (Koren et al. 2016), and polished with nanopolish 0.9.2 (Loman et al. 2015) using 200x coverage of the longest reads aligned back to the assembly with GraphMap 0.5.2 (Sović et al. 2016). Resulting contigs were scaffolded using overlaps detected by nucmer (MUMmer4) (Marçais et al. 2018), which was also used to produce final alignments against the GRCm38 reference.

Copy number analysis

Published FASTQ WGS data for C57BL/6J (SRP001314) and inbred strains (Keane et al. 2011) were downloaded. Reads were clipped of sequencing adapters and quality trimmed using Trimmomatic 0.32 (Bolger et al. 2014) and aligned to the GRCm38 reference using bwa 0.7.10 (Li and Durbin 2010). Read depths were prepared for 500 nt windows across chromosome 13 using samtools (Li et al. 2009) and normalized between strains according to the total number of reads mapping to the chromosome. Ratios of reads mapping within inbred strains in comparison to C57BL/6J were prepared and the median absolute error (MAE) calculated by comparison of these ratios for strains. Log_e_ transformed ratios were plotted in R to compress extremes in the data for visualization.

LTR expression assay

*A Zfp998*–IRES–*GFP* construct was synthesized and inserted into the CMV-driven pcDNA3.1 expression vector by GeneArt (ThermoFisher). Two replication-incompetent viral genomes bearing *mCherry* and *Luciferase*, to be packaged into virions and transduced into recipient cells, were designed to test the expression of pMLV and MoMLV LTRs in the presence and absence of *Zfp998*. To ensure expression of the cassette in the packaging cell culture, the 5’ LTR was modified to include the CMV IE Enhancer (CMV–R–U5, designated LTR*) and the extended packaging signal (Ψ) was included. Final constructs thus had the form LTR*–Ψ–*mCherry*–IRES–*Luciferase*–PPT–LTR. Upon successful infection, the 5’ LTR of integrations would be corrected to the canonical form (U3–R–U5) through the process of reverse transcription. Genomes were synthesized by GeneArt (ThermoFisher) and delivered in the pMS cloning vector. 293T cells cultured in DMEM (Gibco, Waltham, USA) with 10% FBS (BioSera, Nuaille, France) and 1% pen/strep (Sigma-Aldrich, St. Louis, USA) were transfected with either plasmid alongside pczVSVG and pHIT60 (Bock et al. 2000) to supply the components required for virus production. Virion-containing supernatants were harvested and frozen at -80 °C until use. Stocks were titered and 293T cells transduced with standardized amounts of virus to achieve 0.5 MOI. 24 hours later, cells were transfected with either pcDNA3.1-*Zfp998* or with a transfection control (pfEGFPf). Cells were harvested for FACS analysis on Fortessa cell analyzers (Becton Dickinson) after a further 48 hours and levels of mCherry determined. Post-acquisition FACS analysis was conducted with FlowJo 10 (TreeStar) and graphs and statistics produced with Prism 9 (GraphPad).

**References**

Attig J, Young GR, Stoye JP, Kassiotis G. 2017. Physiological and Pathological Transcriptional Activation of Endogenous Retroelements Assessed by RNA-Sequencing of B Lymphocytes. *Front. Microbiol.* 8:2489.

Bock M, Bishop KN, Towers G, Stoye JP. 2000. Use of a Transient Assay for Studying the Genetic Determinants of Fv1 Restriction. *J. Virol.* 74:7422–7430.

Bolger AM, Lohse M, Usadel B. 2014. Trimmomatic: A flexible trimmer for Illumina sequence data. *Bioinformatics* 30:2114–2120.

Evans LH, Morrison RP, Malik FG, Portis J, Britt WJ. 1990. A neutralizable epitope common to the envelope glycoproteins of ecotropic, polytropic, xenotropic, and amphotropic murine leukemia viruses. *J. Virol.* 64:6176–6183.

Ewing B, Green P. 1998. Base-Calling of Automated Sequencer Traces Using Phred. II. Error Probabilities. *Genome Res.* 8:186–194.

Ewing B, Hillier L, Wendl MC, Green P. 1998. Base-Calling of Automated Sequencer Traces Using Phred. I. Accuracry Assessment. *Genome Res.* 8:175–185.

Keane TM, Goodstadt L, Danecek P, White MA, Wong K, Yalcin B, Heger A, Agam A, Slater G, Goodson M, et al. 2011. Mouse genomic variation and its effect on phenotypes and gene regulation. *Nature* 477:289–294.

Kim D, Langmead B, Salzberg SL. 2015. HISAT: a fast spliced aligner with low memory requirements. *Nat. Methods* 12:357–360.

Koren S, Walenz BP, Berlin K, Miller JR, Bergman NH, Phillippy AM. 2016. Canu: scalable and accurate long‐read assembly via adaptive k-mer weighting and repeat separation. *Genome Res.* 27:722–736.

Li H, Durbin R. 2010. Fast and accurate long-read alignment with Burrows-Wheeler transform. *Bioinformatics* 26:589–595.

Li H, Handsaker B, Wysoker A, Fennell T, Ruan J, Homer N, Marth G, Abecasis G, Durbin R. 2009. The Sequence Alignment/Map format and SAMtools. *Bioinformatics* 25:2078–2079.

Liao Y, Smyth GK, Shi W. 2014. FeatureCounts: An efficient general purpose program for assigning sequence reads to genomic features. *Bioinformatics* 30:923–930.

Loman NJ, Quick J, Simpson JT. 2015. A complete bacterial genome assembled de novo using only nanopore sequencing data. *Nat. Methods* 12:733–735.

Love MI, Huber W, Anders S. 2014. Moderated estimation of fold change and dispersion for RNA-seq data with DESeq2. *Genome Biol.* 15:550.

Marçais G, Delcher AL, Phillippy AM, Coston R, Salzberg SL, Zimin A. 2018. MUMmer4: A fast and versatile genome alignment system. *PLoS Comput. Biol.* 14:1–14.

Oliver PL. 1999. Analysis of the control of endogenous murine retrovirus expression by Gv1. Available from: https://discovery.ucl.ac.uk/id/eprint/10103009/

Sović I, Šikić M, Wilm A, Fenlon SN, Chen S, Nagarajan N. 2016. Fast and sensitive mapping of nanopore sequencing reads with GraphMap. *Nat. Commun.* 7:11307.

Wu TD, Watanabe CK. 2005. GMAP: A genomic mapping and alignment program for mRNA and EST sequences. *Bioinformatics* 21:1859–1875.

Young GR, Eksmond U, Salcedo R, Alexopoulou L, Stoye JP, Kassiotis G. 2012. Resurrection of endogenous retroviruses in antibody-deficient mice. *Nature* 491:774–778.
