# Supplementary figures and images for: Gv1, a Zinc Finger Gene Controlling Endogenous MLV Expression
Source: Mol Biol Evol. 2021 Feb 9;38(6):2468–74. doi: 10.1093/molbev/msab039 (PMC8136514; doi:10.1093/molbev/msab039)

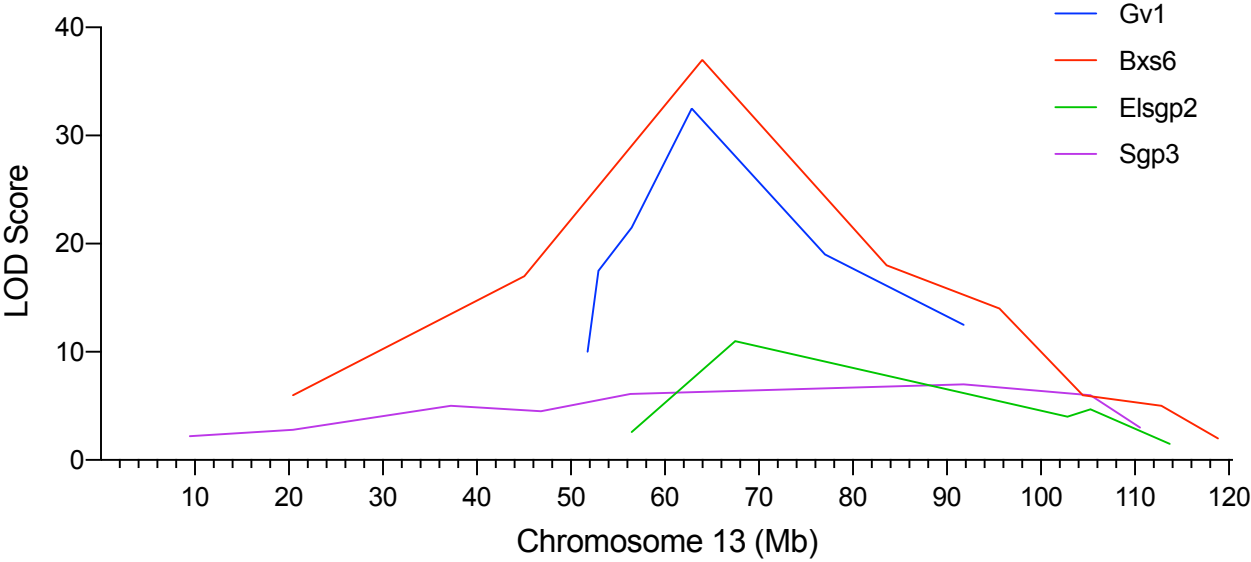

Supplement: msab039_Supplementary_Data [file msab039_supplementary_data.zip › Fig S1.pdf]

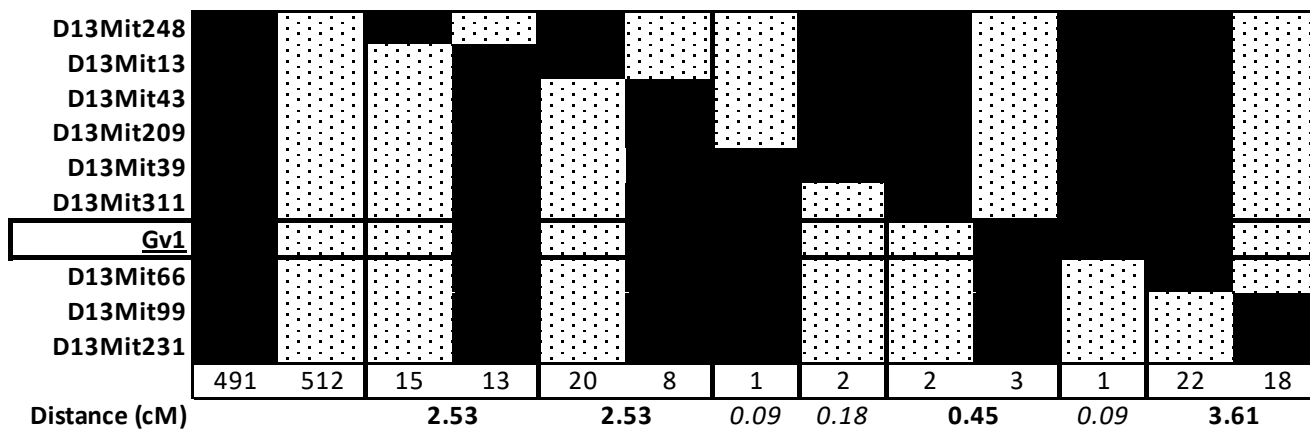

Supplement: msab039_Supplementary_Data [file msab039_supplementary_data.zip › Fig S2.pdf]

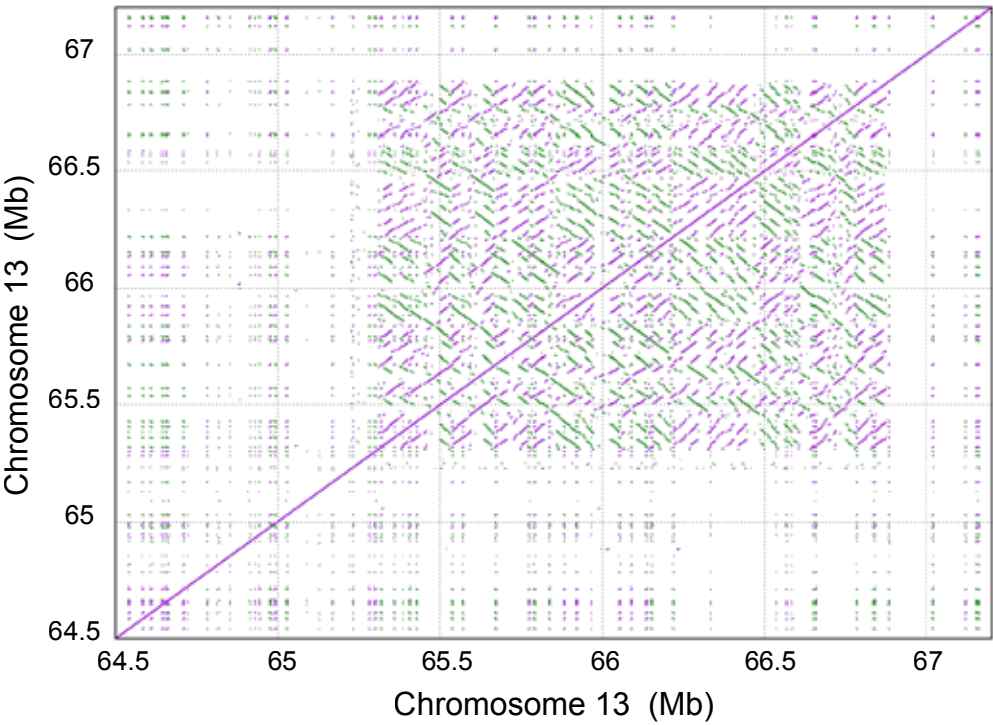

Supplement: msab039_Supplementary_Data [file msab039_supplementary_data.zip › Fig S3.pdf]

Read depth ratio vs B6/J

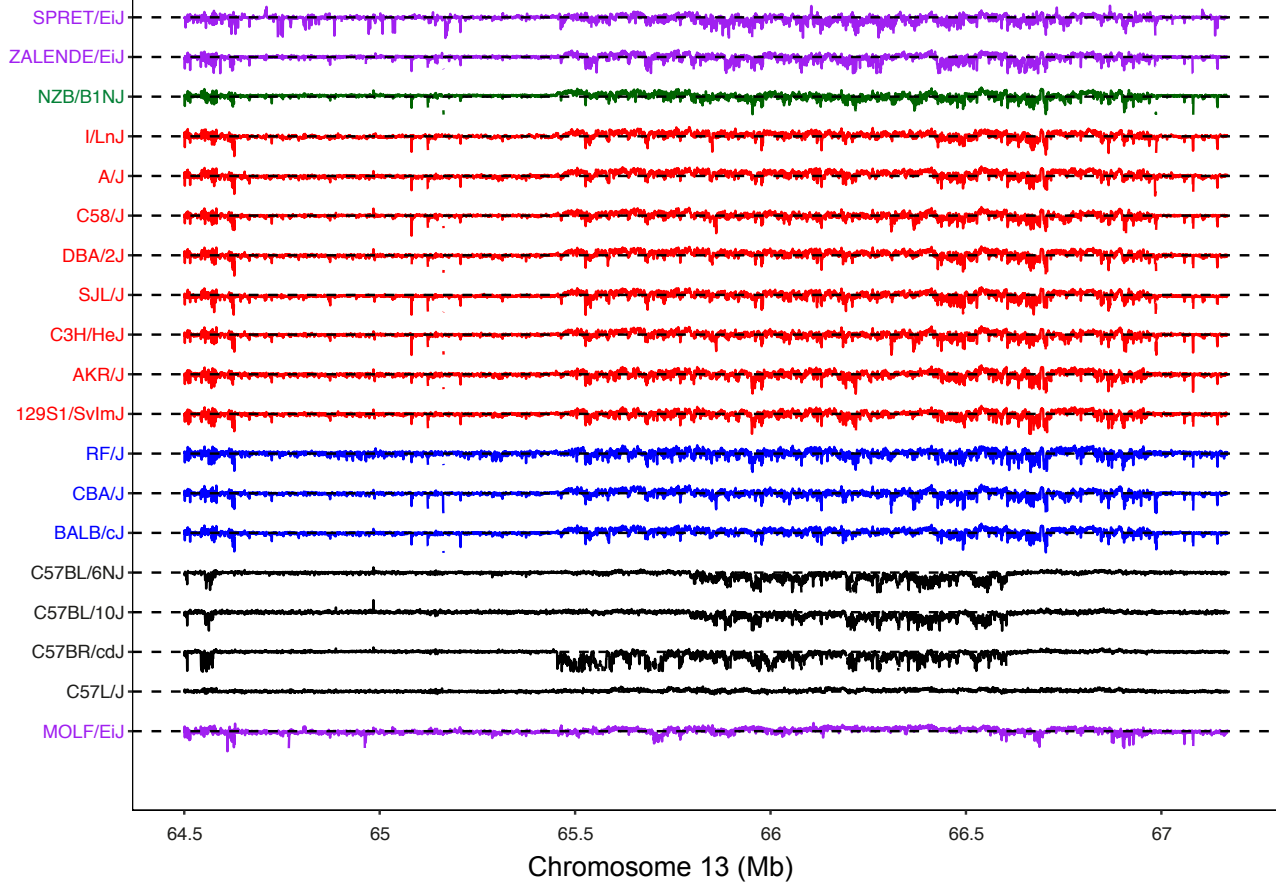

Supplement: msab039_Supplementary_Data [file msab039_supplementary_data.zip › Fig S4.pdf]

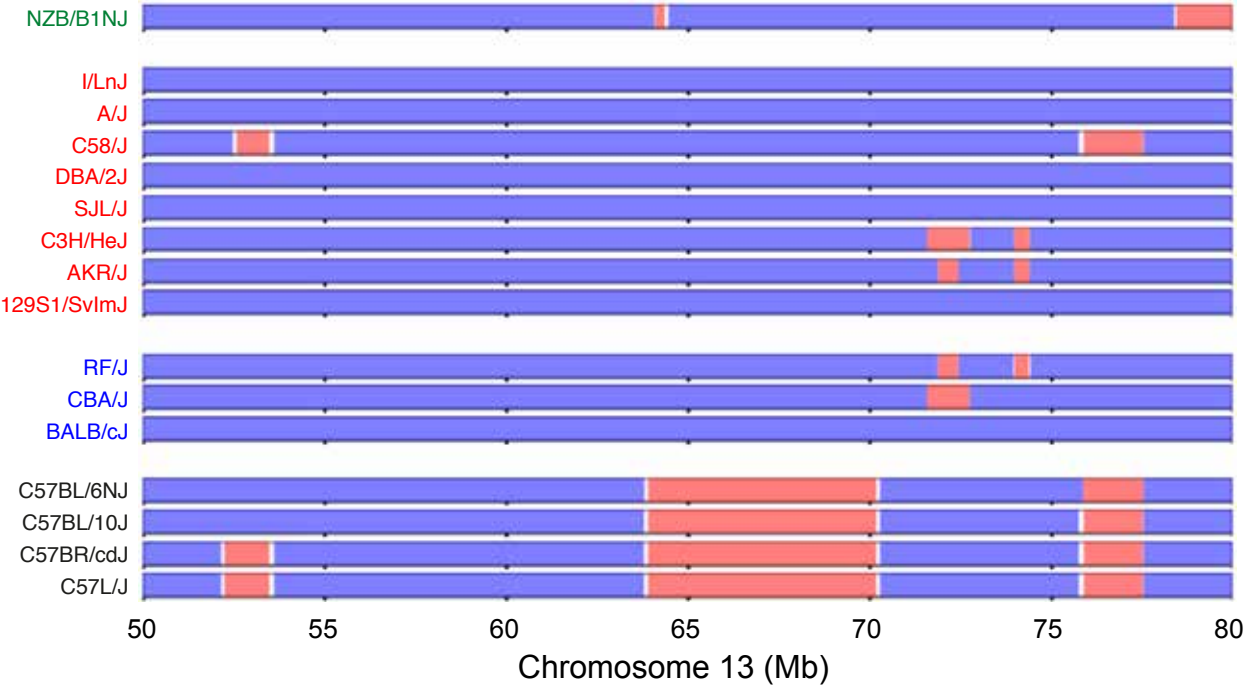

Supplement: msab039_Supplementary_Data [file msab039_supplementary_data.zip › Fig S5.pdf]

Thymus  
Gated on live, CD19<sup>-</sup>

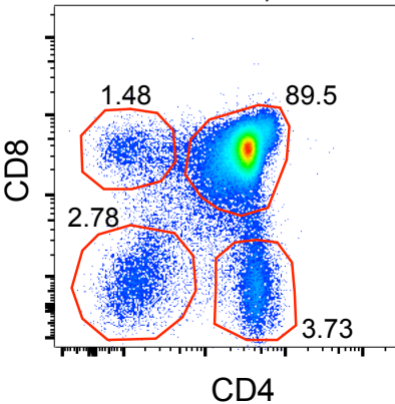

Supplement: msab039_Supplementary_Data [file msab039_supplementary_data.zip › Fig S6.pdf]

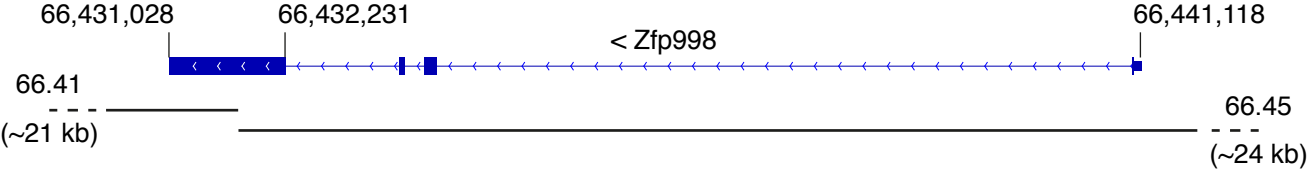

Supplement: msab039_Supplementary_Data [file msab039_supplementary_data.zip › Fig S7.pdf]
